# Hypoxaemia risk in pediatric flexible bronchoscopy for foreign body removal: a retrospective study

**DOI:** 10.1186/s12887-024-04836-6

**Published:** 2024-05-23

**Authors:** Su-Jing Zhang, Min-Yi Lin, Min Zhou, Ying-Zhi Dan, Hong-Bin Gu, Guo-Lin Lu

**Affiliations:** 1 College of Clinical Medicine for Obstetrics & Gynecology and Pediatrics, Fujian Medical University, Department of Anesthesiology, Fujian Children’s Hospital(Fujian Branch of Shanghai Children’s Medical Center), Fujian Key Laboratory of Women and Children’s Critical Diseases ReseFujian Medical Universityarch, Fuzhou, China; 2 Department of Anesthesiology, Fujian Maternity and Child Health Hospital, Affiliated Hospital of Fujian Medical University, Fuzhou, China; 3 Department of Anesthesia, Shanghai Children’s Medical Center, School of Medicine, Shanghai Jiao Tong University, Shanghai, China

**Keywords:** Airway foreign body, Flexible bronchoscopy, Hypoxemia, Children

## Abstract

**Background:**

Hypoxemia represents the most prevalent adverse event during flexible bronchoscopy procedures aimed at foreign body retrieval in pediatric patients; if not expeditiously managed, it carries the potential for cardiac or respiratory arrest. The specific risk factors contributing to the occurrence of hypoxemia during foreign body FB removal via bronchoscopy have yet to be definitively established.

**Methods:**

This retrospective study included a cohort of 266 pediatric subjects from January 1, 2015, to December 31, 2022, who underwent flexible bronchoscopy for the purpose of FB extraction. In this cohort, the supraglottic airway was used to connect the anesthesia apparatus during the removal procedure.

**Results:**

In total, 45 of the pediatric patients (16.9%) experienced episodes of hypoxemia during the FB removal procedure. Multivariate analysis revealed that the following factors were significantly associated with the occurrence of hypoxemia: an operation time exceeding 60 min (odds ratio [OR] 8.55; 95% confidence interval [CI] 3.82–19.13), a maximum diameter exceeding 7 mm (OR 5.03; 95% CI, 2.24–11.29), and the presence of radiological evidence indicating pneumonia (OR 2.69; 95% CI, 1.27–5.69).

**Conclusion:**

During flexible bronchoscopy procedures aimed at FB removal in pediatric patients, there is an increased susceptibility to hypoxemia. Factors including extended operation duration, larger FB dimensions, and radiographic evidence suggestive of pneumonia significantly contribute to a heightened risk of hypoxemia.

## Key message

Factors significantly linked to hypoxemia included an operation time surpassing 60 min, a foreign body with a diameter exceeding 7 mm, and the presence of radiological evidence indicating pneumonia.

**New knowledge**

In pediatric bronchoscopy for foreign body removal, prolonged procedures, larger objects, and radiographic signs of pneumonia increase the risk of hypoxemia.

**Impact**

Hypoxemic risks in rigid bronchoscopy for foreign body removal have been recognized. Supraglottic airway under a controlled ventilation is a new method to support FB removal with a flexible bronchoscopy. However, the associated risks remain unclear. In this study, we delve into the risks of flexible bronchoscopy in pediatric patients, noting prolonged procedures, larger foreign bodies, and pneumonia signs. No cardiac arrest occurred in patients undergoing a controlled ventilation with supraglottic airway. The findings indicate hypoxemia should be reduced, shortening procedures, treating pneumonia, and prioritizing vigilance with large foreign bodies in pediatric patients.

## Introduction

Aspiration from a foreign body (FB) is a frequently encountered incident in children under the age of 3. Failure to promptly address this situation may give rise to life-threatening repercussions. Preventing complications or mortality in early pregnancy requires promptly using bronchoscopy for FB removal [1]. Recently, flexible bronchoscopy has emerged as a prevalent and widely adopted approach for the extraction of foreign bodies [2–4]. Furthermore, the flexibility and exceptional visibility render flexible bronchoscopy a valuable tool for inspecting the distal bronchial regions during surgical procedures.

Hypoxemia is one of the most common adverse events during both flexible bronchoscopy and rigid bronchoscopy. Hypoxic arrest is the primary cause of death during bronchoscopy [1]. Rapid desaturation causes cardiac arrest at a rate of up to 0.36%^5^. It is crucial to identify and focus on the greater risks of hypoxemia among children who undergo bronchoscopy for FB removal. The major factors for hypoxemia during rigid bronchoscopy include young age [5, 6], the organic nature of the FB [7], pneumonia before the procedure [5] and prolonged duration of the procedure [5, 8]. However, the specific risk factors contributing to hypoxemia among children who undergo flexible bronchoscopy for FB removal have not been identified. In this study, we aimed to ascertain the specific risk factors linked to intraoperative hypoxemia in a pediatric population that underwent flexible bronchoscopy.

## Materials and methods

### Study design and approvals

The study was approved by the Ethics Committee of Fujian Provincial Maternity and Children’s Hospital, Affiliated Hospital of Fujian Medical University (Grant No: 2021KLR09069). Written informed consent was waived by the Ethics Committee of our hospital. The study was structured as a retrospective cohort investigation. Information concerning children who underwent flexible bronchoscopy for FB removal was gathered from the Anesthesia Information Management Systems (AIMS) from January 1, 2015, to December 31, 2022.

### Study population

The inclusion criterion included children younger than 18 years with confirmed diagnoses of FB aspiration, as verified through a flexible bronchoscope procedure conducted under general anesthesia using a supraglottic airway (LTLAN; Funia Medical Equipment Co., Ltd., Zhuhai, China). Exclusion criteria comprised children who exhibited preexisting hypoxemia or were on mechanical ventilation before the procedure, those with preexisting cardiopulmonary conditions, cases where the foreign body was located in the larynx, and instances where data records were incomplete.

In patients who underwent multiple bronchoscopy procedures within their hospital stay, only the initial bronchoscopy was considered for analysis. Similarly, if a patient presented with foreign bodies in multiple locations, the measurement of the maximum diameter was considered solely the largest foreign body among those identified.

### Airway management

All procedures were conducted under general anesthesia with a supraglottic airway for controlled ventilation. The size of supraglottic airway was determined by the child’s weight. The middle carrier of supraglottic airway was cut off, and then the supraglottic airway was inserted after the use of muscle relaxant. A flexible pediatric bronchoscope (specifically, the Olympus flexible bronchoscope BF-P 290) was put through the supraglottic airway connected to a swivel. A Y-shaped threaded pipe was linked between the swivel and anesthesia machine. The respiratory was controlled using the volume control mode, utilizing pure oxygen throughout the procedure. The respirator settings were adjusted to maintain end-tidal carbon dioxide (PetCO2) levels between 35 mmHg and 50 mmHg. In instances where SpO2 levels fell below 85%, the flexible bronchoscope was retracted to enhance ventilation function.

### Data sources and measures

Hypoxemia, defined as a pulse oxygen saturation less than 90% as recorded in the Anesthesia Information Management System (AIMS), was observed in children who were normoxic and not on mechanical ventilation before the procedure. The patients received mechanical ventilation via a supraglottic airway during flexible bronchoscopy for FB removal. To mitigate the possibility of “false hypoxemia” due to sensor displacement or patient movement, all the children were monitored using two pulse oximetry probes—one placed on a finger opposite the blood pressure cuff and the other on a toe—throughout the procedure. The stability of heart rate (HR) and pulse oxygen saturation (SpO2) recorded in AIMS for at least 10 s was a criterion for data accuracy (and was not considered an artifact). Blood pressure was logged at five-minute intervals.

Patient data including demographic information (sex, age, weight), FB characteristics (type, duration, location, size), operative details (duration, intraoperative airway hemorrhage), and anesthetic specifics (use of neuromuscular blocking agents) were extracted from medical records. Additionally, radiological findings such as emphysema, pneumonia, atelectasis, and perioperative events including hypoxemia, pneumothorax, bronchial laceration, airway hemorrhage, and reintubation were documented. Reintubation was defined as the first intubation following a supraglottic airway. The primary outcome of interest was the incidence of hypoxemia. These variables were further categorized into subvariables: age in years (divided into < 1, 1–2, 2–3, and ≥ 3); weight in kilograms (kg) (divided into < 10, 10–20, and ≥ 20); FB duration in days (divided into <3, 3–7, 7–30, and ≥ 30); FB type; FB location, maximum FB diameter in millimeters (mm) (divided into ≤ 7, and > 7); operative time in minutes (divided into < 60, and ≥ 60); radiological findings (emphysema, pneumonia, and atelectasis); and use of neuromuscular blockade agents.

### Statistical analyses

Categorical data are depicted as numbers or percentages, while normally distributed quantitative variables are presented as the mean and standard deviation (SD). Nonnormally distributed variables are represented by the median and interquartile range (p25-p75). The relationship between variables and the incidence of hypoxemia was explored through chi-square tests, utilizing Yates correction for two categorical variables and Pearson’s chi-square test for those with multiple categories. When the observed values were less than 5, the chi-square test was substituted for Fisher’s exact test. Variables displaying a *P*-value < 0.1 in the single-factor analysis were advanced for multivariate logistic regression. The outcomes of the regression analyses are presented as odds ratios (ORs) with corresponding 95% confidence intervals (Cis). *P* < 0.05 indicated statistical significance. All the data analysis was conducted using IBM SPSS Statistics for Windows Version 26.0, attributed to IBM Corp, headquartered in Armonk, NY.

## Results

### Characteristics of patients and foreign bodies

**Fig. 1:**
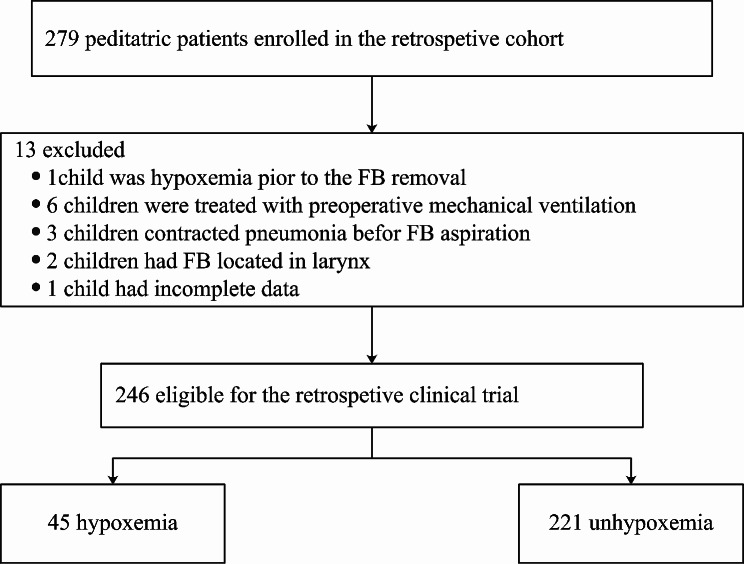
Flow chart illustrating participant involvement, event occurrence, and inclusion analyses

Of the 279 children who underwent flexible bronchoscopy for FB removal between January 2015 and December 2022, specific details were noted: 1 child had preexisting hypoxemia, 6 required preoperative mechanical ventilation, 3 had prior pneumonia related to FB aspiration, 2 had a FB located in the larynx, and 1 had incomplete data. Consequently, 266 children (84 girls, 182 boys) were included in the analysis (Fig. 1). The detailed patient and FB characteristics are summarized in Table 1.

**Table 1:** The characteristics of all children and risk factors associated with hypoxemia

Variables	Participants (*n* = 266)	Hypoxemia (*n* = 45)	Normoxia (*n* = 221)	OR & 95%CI	*P* value
Sex					
Female	84 (31.6%)	16	68	1.24(0.63–2.44)	0.53
Male	182 (68.4%)	29	153	1	-
Age(year)					
<1	14 (5.3%)	1	13	0.34 (0.04–2.7)0	0.471
1–2	185 (69.5%)	34	151	1	-
2–3	46 (17.3%)	10	36	1.23 (0.56–2.73)	0.603
≥ 3	21 (5.7%)	0	21	-	-
Weigh(kg)					
<10	61 (22.9%)	10	51	1	-
10–20	196 (73.7%)	35	161	1.11 (0.51–2.40)	0.793
≥ 20	9 (3.4%)	0	9	-	-
FB location					
Trachea	15 (5.6%)	4	11	2.00 (0.58–6.91)	0.276
Right bronchus	130(48.9%)	20	110	1	-
Left bronchus	119(45.7%)	21	98	1.18 (0.60–2.30)	0.631
Multiple sites	2 (0.8%)	0	2	-	-
FB type					
Nuts	234 (88.0%)	43	191	3.38(0.78–14.7)	0.086
Non-nut	32(12.0%)	2	30	1	-
FB max diameter (mm)					
≤ 7	160 (60.2%)	18	142	1	-
>7	106 (39.8%)	27	79	2.70 (1.40–5.20)	0.002
FB duration (days)					
≤ 5	162 (60.9%)	24	138	1	-
6–30	84(31.6%)	16	68	1.353 (0.68–2.71)	0.394
>30 or unclear	20(7.5%)	5	15	1.917(0.63–5.76)	0.326
Muscle relaxant					
No	25 (9.4%)	4	21	1	-
Mivacurium	91(34.2%)	11	80	0.72 (0.21–2.50)	0.736
Rocuronium	150 (56.4%)	30	120	1.31 (0.42–4.11)	0.789
Operative time (mins)					
<60	209 (78.6%)	21	188	1	
≥ 60	57 (21.4%)	24	33	6.51 (3.26–13.01)	< 0.001
Airway hemorrhage	59 (22.2%)	13	46	1.55(0.75–3.18)	0.235
Radiological findings					
Emphysema	123 (46.2%)	21	102	1.02(0.54–1.94)	0.950
Pneumonia	112 (42.1%)	27	85	2.40 (1.25–4.62)	0.008
Atelectasis	9 (3.4%)	3	6	2.56 (0.62–10.64)	0.181

The median age at diagnosis for FB aspiration was 1.5 years (p25-p75: 1.17-2, range: 0.91-11). The distribution of aspirated FBs revealed 130 children (48.9%) in the right bronchus, 119 children (45.7%) in the left bronchus, 15 children (5.6%) in the trachea, and 2 children (0.8%) with involvement in more than one site. A majority of the FBs (88.0%) were nuts, with the remaining 12.0% categorized as nonnuts. Intervention occurred within 5 days of aspiration or symptom onset for 60.9% (162 children), between 6 and 30 days for 31.6% (84 children), and more than 30 days for 7.5% (20 children). Most FBs had a maximum dimension of ≤ 7 mm, while 39.8% (106 children) had FBs with a maximum dimension exceeding 7 mm. All participants, except for one child who underwent X-ray, received computed tomography (CT) scans before flexible bronchoscopy. Notably, four children had false-negative results in their CT scans.

### Management and outcomes

The procedures had a median duration of 30 min (p25-p75: 18–56), with 21.4% of children enduring procedures lasting ≥ 60 min. Among the patients, 25 (9.4%) underwent flexible bronchoscopy without muscle relaxants, 91 (34.2%) received mivacurium, and 150 (56.4%) were administered rocuronium. Notably, hypoxemia was observed in 45 (16.9%) children, reintubation was required for 32 (12.0%) children, and airway hemorrhage was noted in 59 (22.2%) children. However, pneumothorax, bronchial laceration, and mortality didn’t occured in this study.

### Risk factors associated with hypoxemia

In univariate analysis we identified several clinical factors associated with hypoxemia at a *p*-value < 0.1. These included FB type (OR 3.38; 95% CI 0.78–14.7), a FB with a maximum dimension exceeding 7 mm (OR 2.70; 95% CI 1.40–5.20), pneumonia (OR 2.40; 95% CI 1.25–4.62), atelectasis (OR 2.56; 95% CI 0.62–10.64), and an operation duration equal to or greater than 60 min (OR 6.51; 95% CI 3.26–13.01) (Table 1).

### Multivariate logistic regression analysis

The logistic regression analysis conducted within the study revealed several autonomous predictors of hypoxemia. These included operative durations equal to or exceeding 60 min (OR 8.55; 95% CI, 3.82–19.13), maximum FB dimensions surpassing 7 mm (OR 5.03; 95% CI, 2.24–11.29), and pneumonia (OR 2.69; 95% CI, 1.27–5.69) (Table 2).

**Table 2:** Logistic regression of factors associated with hypoxemia

Variables	OR & 95%CI	*P* value
FB type	1.84 (0.39–8.67)	0.332
FB size	5.03 (2.24–11.29)	< 0.001
Operative time (≥ 60 min)	8.55 (3.82–19.13)	< 0.001
Radiological findings		
Pneumonia	2.69 (1.27–5.69)	0.010
Atelectasis	3.53 (0.62-20.00)	0.154

## Discussion

In this study, hypoxemia emerged as a prevalent complication during fiberoptic bronchoscopy for FB removal under general anesthesia with supraglottic airway. Additionally, risk factors linked to hypoxemia were pinpointed, including an operation duration equal to or exceeding 60 min, a maximum FB dimension exceeding 7 mm, and the presence of pneumonia. Notably, other complications such as airway hemorrhage and the need for reintubation were observed. Fortunately, there were no reported incidents of pneumothorax, bronchial laceration, or mortality.

Untreated hypoxemia during fiberoptic bronchoscopy can potentially lead to severe oxygen deprivation and subsequent cardiac arrest. Specifically, the data revealed that 37 patients (0.36%) experienced cardiac arrest attributed to rapid desaturation [7]. Several factors contributed to this observation. First, patients with FBs undergoing procedures face an increased risk of perioperative respiratory adverse events linked to conditions such as pneumonia, atelectasis, and emphysema. Second, the process of flexible bronchoscopy itself can induce airway injuries, such as glottic edema and hemorrhage. The bronchoscope can induce airway closure, laryngo-bronchial spasms, and trap mucosal secretions, leading to bleeding-prone granulation tissue. Vigorous suctioning exacerbates the reduction in airways, alveolar volume, and oxygen concentration, contributing to hypoxemia. Third, the presence of a foreign body within the airway can prompt partial or complete obstruction, impairing pulmonary ventilation. Moreover, FB aspiration, which is notably prevalent in children under 2 years old, amplifies the risk due to reduced functional reserve capacity and heightened oxygen consumption. These combined factors significantly heighten the risk of hypoxemia during fiberoptic bronchoscopy.

According to Chen et al., the hypoxemia rate during rigid bronchoscopy for FB removal was 9.2% among patients utilizing manual jet ventilation [6]. Consistent with the findings in a prior study [9], this rate notably increased to 25.5% in patients undergoing the procedure under spontaneous ventilation [5]. The rate was 23.4% with manual intermittent positive pressure ventilation [6]. In this study, the incidence of fiber bronchoscopy utilizing a supraglottic airway was 16.9%, demonstrating a lower rate compared to rigid bronchoscopy except in cases involving manual jet ventilation. Furthermore, no cardiac arrest occurred. Controlled ventilation must be improved to reduce hypoxemia rate, optimization of respiratory parameters, maintenance of airway impermeability and treatment of throat swelling. Hypoxemia has been associated with various factors during rigid bronchoscopy for FB removal, including patient age, foreign body nature, surgery duration, preprocedural pneumonia, and ventilation mode [5]. Our findings affirm the association of intraoperative hypoxemia with operative duration, foreign body size, and pneumonia incidence. Thus, hypoxemia incidence can be decreased by reducing the operation duration. However, we must realize that frequent interruptions in bronchoscopy may allow for improved oxygenation and ventilation of hypoxemic patients, special in early cases. It is important to improve controlled ventilation management to reduce hypoxemia for a less duration. Treatment for preprocedural pneumonia might reduce the risk of hypoxemia. It must be emphasized that FB removal should be conducted without delay even for treating pneumonia.

The incidence of hypoxemia incidence is documented to increase with prolonged FB removal [5, 6, 9]. In our earlier study, we similarly identified an extended operation duration as a risk factor for reintubation [10]. The extended duration of the operation could have heightened the risk of airway trauma and secretion buildup. Additionally, a longer operation time necessitates prolonged exposure to anesthetic drugs, increasing the likelihood of hypoxemia. Studies indicate that hypoxemia tends to manifest during rigid bronchoscopy for FB removal in procedures lasting more than 20 min [5]. In this study, procedures exceeding 60 min demonstrated an increased risk of hypoxemia. This could be attributed in part to the controlled ventilation via the supraglottic airway during bronchoscopy.

Most of the FBs identified were organic, predominantly nut-based foods such as peanuts and seeds. These food types possess the capacity to absorb fluids, potentially exacerbating obstructive processes [7]. Nut oils can induce localized inflammation, airway edema, and granulation tissue formation, all of which can exacerbate the severity of bronchial obstruction. A study illustrated that children who had aspirated plant seeds exhibited a notably greater risk of hypoxemia (*P* = 0.02, OR = 2.65) than did those who had not [5]. However, in the study by Bittencourt PF, the nature of the FB had no direct impact on hypoxemia [8]. Consistently, in our findings, the organic nature of the treatment alone did not indicate an increased risk of hypoxemia. Instead, we observed an association between a foreign body with a maximum diameter exceeding 7 mm and the occurrence of hypoxemia. This situation can arise from partial or complete obstruction of the airway during bronchoscopy. Another plausible explanation is that foreign bodies with cylindrical or spherical shapes might obstruct the airway, leading to a greater likelihood of dislodgment as they pass through the glottic opening; this, in turn, could prolong surgical procedures and heighten the risk of anesthesia-related complications [11].

The presence of FBs within the bronchial tree can prompt irreversible pulmonary alterations via mucosal inflammation, culminating in conditions such as bronchial stenosis, emphysema, pneumonia, and atelectasis. Recently, CT scanning has shown heightened sensitivity and specificity in detecting FBs, providing an advantage by revealing the characteristics of suspected FBs. In this study, a majority of the pediatric patients exhibited a combination of emphysema, pneumonia, and atelectasis, as identified through CT scans. Aligned with the findings of other studies [12], emphysema has emerged as a prevalent condition among children with FB. However, it’s not regarded as a direct risk factor for hypoxemia. The pneumonia rate observed in our study (42.1%) mirrored that reported in a previous study (48.63%). Notably, pneumonia emerged as a substantial risk factor for hypoxemia in our investigation, a finding consistent with observations during rigid bronchoscopy for FB removal [7]. Previous studies have shown evident the association between secondary inflammatory processes and the occurrence of hypoxemia [13].

Acknowledging the limitations of our study is imperative. First, this was a retrospective, single-center, cross-sectional investigation reliant on the accuracy of clinical data obtained from medical records. Second, the study may have overlooked instances of transient hypoxemia, potentially leading to an underestimation of its incidence. Third, the exact cause of hypoxemia, such as severe glottic edema, airway spasms, or compromised airway ventilation, could not be specifically identified. Fourth, while efforts were made to control for confounding factors, the influence of anesthesiologists’ and surgeons’ experience could not be entirely mitigated. Finally, we did not analyze potential factors associated with hypoxemia, such as ventilation and anesthesia modes, due to the retrospective nature of the research.

## Conclusion

Our findings highlight that an extended operation duration (≥ 60 min), a larger foreign body size (diameter > 7 mm), and the presence of pneumonia according to radiological findings are correlated with an increased risk of hypoxemia. It is pivotal to screen high-risk children and implement suitable pretreatment measures to mitigate the risk of hypoxemia during fiber optic bronchoscopy procedures.

## Data Availability

No datasets were generated or analysed during the current study.
